# Altered Activation of Innate Immunity Associates with White Matter Volume and Diffusion in First-Episode Psychosis

**DOI:** 10.1371/journal.pone.0125112

**Published:** 2015-05-13

**Authors:** Teemu Mäntylä, Outi Mantere, Tuukka T. Raij, Tuula Kieseppä, Hanna Laitinen, Jaana Leiviskä, Minna Torniainen, Lauri Tuominen, Outi Vaarala, Jaana Suvisaari

**Affiliations:** 1 Mental Health Unit, National Institute for Health and Welfare, Helsinki, Finland; 2 Brain Research Unit, O.V. Lounasmaa Laboratory and Advanced Magnetic Imaging Centre, Aalto NeuroImaging, Aalto University School of Science, Espoo, Finland; 3 Institute of Behavioural Sciences, University of Helsinki, Helsinki, Finland; 4 Department of Psychiatry, Helsinki University Central Hospital, Helsinki, Finland; 5 Institute of Clinical Medicine, Department of Psychiatry, Helsinki University, Helsinki, Finland; 6 Vaccination Programme Unit, National Institute for Health and Welfare, Helsinki, Finland; 7 Genomics and Biomarkers Unit, National Institute for Health and Welfare, Helsinki, Finland; 8 Department of Clinical Neuroscience, Karolinska Institutet, Stockholm, Sweden; 9 Turku PET centre, University of Turku, Turku, Finland; 10 Department of Psychiatry, University of Turku, Turku, Finland; 11 Respiratory, Inflammatory and Autoimmunity, Innovative Medicine, AstraZeneca R & D, Mölndal, Sweden; 12 Institute of Clinical Medicine, University of Helsinki, Helsinki, Finland; King's College London, UNITED KINGDOM

## Abstract

First-episode psychosis (FEP) is associated with inflammatory and brain structural changes, but few studies have investigated whether systemic inflammation associates with brain structural changes in FEP. Thirty-seven FEP patients (median 27 days on antipsychotic medication), and 19 matched controls were recruited. Serum levels of 38 chemokines and cytokines, and cardiovascular risk markers were measured at baseline and 2 months later. We collected T1- and diffusion-weighted MRIs with a 3 T scanner from the patients at baseline. We analyzed the association of psychosis-related inflammatory markers with gray and white matter (WM) volume using voxel-based morphometry and WM diffusion using tract-based spatial statistics with whole-brain and region-of-interest (ROI) analyses. FEP patients had higher CCL22 and lower TGFα, CXCL1, CCL7, IFN-α2 and ApoA-I than controls. CCL22 decreased significantly between baseline and 2 months in patients but was still higher than in controls. The association between inflammatory markers and FEP remained significant after adjusting for age, sex, smoking and BMI. We did not observe a correlation of inflammatory markers with any symptoms or duration of antipsychotic treatment. Baseline CCL22 levels correlated negatively with WM volume and positively with mean diffusivity and radial diffusivity bilaterally in the frontal lobes in ROI analyses. Decreased serum level of ApoA-I was associated with smaller volume of the medial temporal WM. In whole-brain analyses, CCL22 correlated positively with mean diffusivity and radial diffusivity, and CXCL1 associated negatively with fractional anisotropy and positively with mean diffusivity and radial diffusivity in several brain regions. This is the first report to demonstrate an association between circulating chemokine levels and WM in FEP patients. Interestingly, CCL22 has been previously implicated in autoimmune diseases associated with WM pathology. The results suggest that an altered activation of innate immunity may contribute to WM damage in psychotic disorders.

## Introduction

Psychotic disorders are associated with immunological changes, some of which persist beyond remission of psychotic symptoms [1]. These changes include elevations of inflammatory cytokines and chemokines in blood and cerebrospinal fluid (CSF), alterations in monocyte and T-cell activation, and increased gene expression of pro-inflammatory genes in peripheral blood cells, CSF as well as in post-mortem brain tissue [1–9]. Many of these alterations are evident already in first-episode psychosis (FEP) [1,10,11]. Several studies suggest that cells of the mononuclear phagocyte system, i.e. monocytes, dendritic cells and macrophages, might be especially relevant in the etiology of psychosis [2,3,6]. The balance between different T-cell populations also seems to be altered [9]. The largest genome-wide association study of schizophrenia to date shows further support for the role of immunological factors in the pathogenesis of symptoms: genes expressed in the immune cells, such as STAT6 and TCF4, were overrepresented in the genetic loci associated with schizophrenia [12]. In addition, some non-steroidal anti-inflammatory drugs are effective as adjuvant therapy in treating psychotic symptoms in patients with schizophrenia [13]. However, peripheral immunological alterations may reflect several factors associating with psychotic disorders [14,15], including weight gain [16], metabolic syndrome [17,18] and antipsychotic medication [19,20].

Schizophrenia is related to widespread structural brain changes and functional connectivity deficits [21–25]. Meta-analyses of voxel-based morphometry studies in psychotic disorders show pervasive gray matter (GM) changes [26–30]. In the white matter (WM), meta-analyses of diffusion tensor imaging (DTI) studies indicate differences particularly in the medial frontal lobes, including the cingulum bundle and the interhemispheric connections through the corpus callosum, and temporal lobes, and these differences are already seen after the first psychotic episode [31–33]. Factors contributing to these changes in psychosis are largely unknown. However, in other neuropsychiatric and neurodegenerative diseases as well as in cognitive decline related to aging, both peripheral immunological alterations and microglia activation seem to contribute to brain structural changes [34–38].

Converging evidence suggests that neuroinflammation is important in schizophrenia [39,40]. According to a recent systematic review, this is supported by neuropathological studies finding higher microglial density in subjects with schizophrenia than in controls in several brain areas, particularly in the WM [40]. Also, positron emission tomography imaging studies have found increased microglial activation in schizophrenia [41–43]. Moreover, one DTI study implicated that neuroinflammation could explain changes in WM diffusion in first-episode schizophrenic patients [44].

The role of peripheral inflammation in WM pathology is, however, not known. Peripheral cytokines and chemokines can affect the central nervous system in several ways: via the hypothalamic-pituitary-adrenal axis, the autonomic nervous system, and even directly by crossing the blood-CSF barrier via the choroid plexus [14,45–50]. Furthermore, elevations in peripheral cytokines or other immunological markers have been shown to be correlated with WM changes in multiple sclerosis [51], in age-related cognitive decline [38], and in late-life depression associated with cognitive impairment [52]. Some recent studies suggest that systemic inflammation may contribute to brain structural changes also in psychotic disorders. A study conducted in FEP patients found that increased interleukin-6 (IL-6) gene expression in leukocytes correlated with smaller left hippocampal volume [53], while another study conducted in young adults with schizophrenia found a correlation between peripheral IL-6 and C-reactive protein (CRP) levels and WM diffusion [54]. In addition, an aggregate measure of pro-inflammatory cytokines predicted progressive right prefrontal cortex gray matter thinning in individuals at clinical high risk for psychosis, particularly in those who transitioned to psychosis during follow-up [55]. However, previous studies have not, to our knowledge, investigated associations between peripheral inflammatory markers and brain volumetric and diffusion measures in FEP.

Our hypothesis was that peripheral immunological and metabolic alterations associate with changes in brain morphology in FEP patients. To map immunological and metabolic alterations in FEP, we used a comprehensive set of 38 cytokines and chemokines as well as cardiometabolic markers. Then, we investigated whether changes in the systemic inflammatory and metabolic markers in FEP associate with brain morphology. We hypothesized that the serum markers that were higher in the patient group than in the control group would correlate negatively with GM volume, WM volume and fractional anisotropy measures and positively with mean diffusivity and radial diffusivity measures, while the markers that were lower in the patient group would have the opposite effect.

## Materials and Methods

### Clinical study protocol

The ongoing study started on November 2010. Patients aged 18 to 40 years with first contact with psychiatric care for psychosis were recruited from the area of the Hospital District of Helsinki and Uusimaa. Psychosis was defined as receiving a score of at least 4 in the items assessing delusions or hallucinations in the Brief Psychiatric Rating Scale (BPRS). All patients with primary psychotic disorders were included. Patients with FEP were assessed three times. The baseline assessment was conducted as soon as the patient had entered treatment and was able to give informed consent, and the follow-ups were conducted at 2 and 12 months. The methods used in the clinical assessment are described in detail in S1 Table. Briefly, the severity of positive and negative psychotic symptoms (current and worst period) [56], current symptoms of mania [57], depression [58], anxiety [59], obsessive-compulsivity [60], and harmful alcohol use [61] were evaluated. Diagnostic assessment was done at 2 months and 1-year follow-up based on the Research Version of The Structured Clinical Interview for DSM-IV Axis I Disorders (SCID-I)–interview and all information from medical records, and the diagnosis was done by a senior psychiatrist (JS) together with the interviewer. In case of uncertainty, a consensus diagnosis between the senior psychiatrists (JS, OM, TK) was made. Data were also gathered on sociodemographic factors, functioning, family history of psychiatric disorders, medication, substance use, physical activity, diet and smoking, and the interviewer measured weight, height, blood pressure and waist circumference (See Table 1).

**Table 1:** Baseline sociodemographic and clinical characteristics of the sample including cases (*n* = 37) and controls (*n* = 19).

	FEP patients^a^	Controls	
	n (%), or median (25%, 75%)	n (%), or median (25%, 75%)	*p*
Age	26.1 (21.9, 28.0)	27.0 (23.7, 33.9)	0.11
Male	21/37 (56.8%)	10/19 (52.6%)	0.77
Living with parents	10/37 (27.0%)	1/19 (5.3%)	0.052
No vocational or higher education	22/37 (59.6%)	2/19 (10.5%)	0.001 (Fisher)
Employed, military or student	17/37 (45.9%)	18/19 (94.7%)	0.04 (Fisher)
BMI	22.2 (21.1, 24.4)	23.5 (20.9–25.3)	0.50
Current smoking	11/29 (37.9%)	2/19 (10.5%)	0.049 (Fisher)
Lifetime smoking	18/30 (48.6%)	6/19 (31.6%)	0.053
No substance use lifetime^b^	26/37 (70.3%)	18/19 (94.7%)	0.043 (Fisher)
Active in sports min. 1h weekly	27/30 (90.0%)	18/19 (94.7%)	0.18 (Fisher)
Family history of psychiatric disorders	22/35 (62.9%)	2/17 (11.8%)	0.001 (Fisher)
AUDIT	5.8 (2, 12.5)	6 (3, 12)	0.65
SOFAS	40 (35, 40)	90 (85, 90)	<0.001
GAF	32 (30, 38)	90 (85, 90)	<0.001
BDI	10.5 (0–43)	0 (0–31)	<0.001
BAI	16 (0–49)	2 (0–14)	<0.001
OCI-R	13 (0–42)	3 (0–16)	<0.001
MDQ screen positive	7/30 (18.9%)	1/19 (5.3%)	0.03 (Fisher)

^a^ Diagnosis Schizophrenia (*n* = 19), Schizophreniform disorder (*n* = 4), BD with psychotic features (*n* = 3), MDD with psychotic features (*n* = 1), Psychotic disorder NOS (*n* = 1), Schizoaffective disorder (*n* = 2), Substance-induced psychotic disorder (*n* = 1, Magnetic resonance images were not available from this participant), Delusional disorder (*n* = 1).

^b^ Substance use does not include alcohol, nicotine or caffeine.

Abbreviations: AUDIT, the alcohol use disorders identification test; BAI, Beck anxiety inventory; BDI, Beck depression inventory; BMI, body mass index; GAF, global assessment of functioning scale; MDQ, mood disorder questionnaire; OCI-R, obsessive-compulsive inventory revised; SOFAS, social and occupational functioning assessment scale.

Controls, matched by age, sex and region of residence, were identified from the Population Register Center and assessed with the same protocol as the patients. The exclusion criteria for the controls were a lifetime history of psychotic disorder, any chronic neurological, endocrinological, or cardiovascular disease, and any condition that prevents Magnetic Resonance Imaging (MRI).

From this analysis, we excluded people with diagnosed diabetes (*n* = 3).

### Ethics statement

The study protocol was approved by the Ethics Committee of the Hospital District of Helsinki and Uusimaa (257/12/03/03/2009) and by the institutional review boards of the National Institute for Health and Welfare, Helsinki, Finland, and the University of Helsinki, and all participants gave a written informed consent. Patient’s capacity to give informed consent was assessed by the treating psychiatrist.

### Laboratory analytical methods

A fasting blood sample was collected in the next morning after the interview at 8 to 10 am. Serum and plasma samples were immediately aliquoted and stored at -80°C. Serum total cholesterol, high-density lipoprotein (HDL) cholesterol, triglycerides, apolipoprotein A-I (ApoA-I) and B (ApoB) and plasma glucose were measured with enzymatic by Abbott Architect ci8200 analyzer (Abbott Laboratories, Abbott Park, IL, USA) in the laboratory of the Disease Risk Unit at National Institute for Health and Welfare. Apolipoprotein A-I (ApoA-I) and B were determined with immunoturbimetric assays (Abbott) and hs-CRP with latex turbidometric immunoassay (Sentinel, Milan, Italy). Insulin and C-peptide were measured with chemiluminescent microparticle immunoassays (Abbott). Low-density lipoprotein (LDL) cholesterol was calculated by the Friedewald formula. The mean inter-assay coefficient of variations (CVs) for cholesterol, HDL cholesterol, triglycerides, and glucose were 1.0%, 2.2%, 1.5%, and 1.4%. The mean CV%s for ApoA-I, ApoB, hs-CRP, insulin, and C-peptide were 1.8%, 2.0%, 4.3%, 2.4%, and 2.5%, respectively.

The levels of serum inflammatory markers were studied at baseline and at 2 months using the Milliplex MAP Kit (HCYTMAG-60K-PX38, Millipore Corp., Billerica, MA) including 38 cytokines and chemokines listed in Table 2. Quantification of the inflammatory markers was carried out with Magpix instrument and xPONENT 4.2 software (Luminex Corp., Austin, TX). Concentration of each marker was determined from an 8-point dilution standard curve using five parameter logistic regression. Samples with concentration below the minimum detectable concentration (MinDC) were given a value equal to MinDC, which was determined for each marker individually as the lowest concentration on the standard curve’s linear phase (S2 Table).

**Table 2:** Differences between cases (*n* = 37) and controls (*n* = 19) in metabolic and inflammatory factors.

		Case		Control		
	**Measure**	**Median** ^**a**^	**25% and 75%**	**Median** ^**a**^	**25% and 75%**	***p***
hs-CRP	mg/l	0.61	0.29, 2.6	0.93	0.38, 2.5	0.72
Glucose	mmol/l	M 4.26	3.96, 4.51	4.32	3.92, 4.56	1.00
Insulin	mU/l	8.1	5.9, 16.9	7.2	4.2, 9.7	0.08
C-peptide	nmol/l	549.0	433.5, 871.5	458.0	463.0, 621.0	0.06
**ApoA-I**	g/l	**1.32**	**1.21, 1.40**	**1.38**	**1.33, 1.66**	**0.023**
ApoB	g/l	M 0.79	0.62, 0.89	0.67	0.55, 0.85	0.22
Cholesterol	mmol/l	4.46	4.08, 5.25	4.49	3.81, 5.70	0.92
HDL-C	mmol/l	1.33	1.11, 1.50	1.37	1.30, 1.60	0.11
LDL-C	mmol/l	2.79	2.38, 3.30	2.52	2.09, 3.18	0.36
Triglycerides	mmol/l	1.1	0.77, 1.4	0.85	0.68, 1.1	0.11
**Innate immune system cytokines**						
**IFN-α2**	**pg/ml**	**24.8**	**16.7, 40.2**	**37.8**	**24.8, 67.7**	**0.027**
IL-1a	pg/ml	32.4	3.2, 85.6	42.7	3.2, 117.8	0.78
IL-1ra	pg/ml	24.4	15.5, 44.1	27.1	14.5, 61.4	0.61
IL-1β	pg/ml	1.5	0.8, 12.4	2.8	0.8, 12.0	0.84
IL-6	pg/ml	2.3	1.3, 10.7	7.2	1.3, 20.6	0.23
TNF**-**α	pg/ml	10.3	7.2, 13.8	10.4	7.5, 12.6	0.91
**Th1 cytokines**						
IFN-γ	pg/ml	17.6	7.4, 57.5	21.1	12.3, 110.1	0.21
IL-12p40	pg/ml	24.3	7.4, 56.3	21.7	7.4, 79.5	0.79
IL-12p70	pg/ml	5.8	2.8, 11.3	15.0	4.4, 10.7	0.48
**Th2 cytokines**						
IL-4	pg/ml	13.5	4.5, 55.1	32.4	4.5, 63.2	0.45
IL-5	pg/ml	1.3	1.3, 1.5	1.4	1.3, 3.3	0.19
IL-13	pg/ml	1.6	1.3, 21.1	14.8	1.3, 40.6	0.24
**Th17 cytokines**						
IL-17	pg/ml	7.8	1.6, 17.2	7.4	2.6, 29.1	0.32
**Regulatory T-cell cytokines**						
IL-10	pg/ml	8.1	1.5, 25.3	18.8	1.3, 56.5	0.60
**Other cytokines**						
IL-2	pg/ml	1.4	1, 16.0	5.2	1, 23.8	0.44
IL-3	pg/ml	1.3	1.3, 2.4	3.6	1.3, 3.5	0.37
IL-7	pg/ml	9.9	6.8, 12.8	10.2	6.9, 14.2	0.62
IL-8	pg/ml	14.5	10.4, 19.4	10.2	6.9, 14.2	0.83
IL-9	pg/ml	1.2	1.2, 6.3	3.5	1.2, 11.6	0.32
IL-15	pg/ml	2.2	1.3, 15.8	2.6	1.3, 20.3	0.76
**Chemokines**						
CCL2	pg/ml	422.8	352.6, 367.2	430.3	337.8, 515.8	0.78
CCL3	pg/ml	9.3	4.0, 14.8	8.8	2.9, 14.9	0.80
CCL4	pg/ml	44.8	25.4, 60.0	42.1	28.0, 82.9	0.94
**CCL7**	pg/ml	**13.1**	**5.8, 21.0**	**24.7**	**9.4, 76.1**	**0.010**
CCL11	pg/ml	166.1	137.7, 221.7	137.5	113.5, 164.8	0.052
**CCL22**	pg/ml	**1864.8**	**1467.3, 2244.7**	**1210.8**	**1119.1, 1498.9**	**0.0011**
**CXCL1**	pg/ml	**571.6**	**474.4, 693.9**	**768.6**	**619.5, 988.3**	**0.0016**
CX3CL1	pg/ml	74.7	46.4, 136.8	95.5	49.2, 179.1	0.29
CXCL10	pg/ml	292.4	224.5, 382.8	322.7	224.5, 383.8	0.22
**Other inflammation related markers**						
**TGFα**	**pg/ml**	**3.1**	**2.0, 4.7**	**5.3**	**3.7, 13.3**	**0.0042**
EGF	pg/ml	113.8	64.2, 153.4	124.5	72.2, 172.3	0.51
FGF-2	pg/ml	46.1	34.2, 68.6	46.3	37.8, 70.0	0.62
FLT-3L	pg/ml	5.4	5.4, 11.2	7.5	5.4, 26.0	0.24
G-CSF	pg/ml	55.0	40.5, 82.4	49.7	30.9, 76.8	0.55
GM-CSF	pg/ml	20.4	14.9, 33.8	22.4	17.7, 46.7	0.14
TNF-β	pg/ml	5.6	1.5, 24.7	14.5	1.5, 46.8	0.31
VEGF	pg/ml	161.8	84.0, 213.7	148.5	99.4, 260.7	0.48
sCD40L	pg/ml	52087.2	26736.7, 61768.8	49626.2	23785.0, 78774.1	0.91

^a^ If not indicated, we present medians for non-normally distributed continuous or ordinal variables; means as indicated by M and SD are presented for normally distributed variables. Abbreviations: Apo, apolipoprotein; CCL, chemokine (C-C motif) ligand; CXCL, Chemokine (C-X-C motif) ligand; EGF, epidermal growth factor; FGF, fibroblast growth factor; FLT-3L, Fms-related tyrosine kinase 3 ligand; G-CSF, Granulocyte-colony stimulating factor; GM-CSF, granulocyte-macrophage colony-stimulating factor; HDL-C, high density lipoprotein cholesterol; hs-CRP, high sensitivity C-reactive protein; IFN, interferon; IL, interleukin; LDL-C, Low Density Lipoprotein cholesterol; sCD40L, soluble CD40 Ligand; TGF, transforming growth factor; TNF, tumor necrosis factor.

### Brain imaging

We collected structural T1-weighted MRI and DTI at baseline first using a Signa VH/i 3 T scanner (GE Healthcare, Chalfont St Giles, UK) with a 16-channel coil, and then, due to update of the scanner in the research centre, using Magnetom Skyra 3 T system and a 32-channel head coil (Siemens AG, Erlangen, Germany) at Aalto AMI Centre, Aalto NeuroImaging, Aalto University School of Science. Established sequences were used, and the detailed parameters are described in Table 3. With the GE scanner, we used a spoiled-gradient-echo sequence to acquire the T1-weighted images in 180 slices with 1.02x1.02x1 mm voxels. With the Siemens scanner, a magnetization-prepared rapid gradient echo sequence was used for 176 sagittal/192 transversal slices with 1x1x1 mm voxels. DTI data were acquired during the same imaging session. With the GE scanner, diffusion sensitizing gradients (*b* = 1000 s/mm^2^) were used to image 60 noncollinear directions and 4 non-diffusion weighted images (*b* = 0 s/mm^2^) in 56 axial slices without gaps and with voxel size of 1.88x1.88x3 mm. With the Siemens scanner, the same *b*-values were used in a 2-dimensional spin-echo EPI sequence. Sixty-four noncollinear directions and 1 non-diffusion weighted image were gathered in 58 axial slices without gaps; voxel size was 1.88x1.88x3 mm.

**Table 3:** Brain imaging parameters.

Parameter	Scanner			
	GE		Siemens	
Sequence	T1	DTI	T1	DTI
TR (ms)	10	10,000	2530	9500
TE (ms)	3	100	3.3–3.75	81
flip angle (degrees)	15	90	7	90
matrix size	256x256	128x128	256x256	128x128
field of view (cm)	26	24	25.6	24
voxel dimensions (mm^3^)	1.02x1.02x1	1.88x1.88x3	1x1x1	1.88x1.88x3

Abbreviations: DTI, diffusion tensor imaging; TE, echo time; TR, repetition time.

### Preprocessing of structural MRI data

The T1-weighted MRIs were analysed with the SPM8 software (http://www.fil.ion.ucl.ac.uk/spm/software/spm8/), and VBM8 toolbox (http://dbm.neuro.uni-jena.de/vbm8/) for voxel-based morphometry [62]. The images were reoriented manually and bias corrected for intensity non-uniformities. An adaptive maximum a posteriori-method was used for segmentation of the images to GM, WM, and CSF, followed by partial volume estimation and denoising. The segmented images were normalized to MNI-space using high-dimensional nonlinear DARTEL normalization [63], and smoothed with a Gaussian kernel (full width at half maximum = 8 mm). In order to estimate regional brain volume, nonlinear modulation was applied to the images after normalization (http://dbm.neuro.uni-jenade/vbm/segmentation/modulation/) [64].

### Preprocessing of DTI data

Brain WM diffusion was studied with DTI. Fractional anisotropy is the most used indicator of WM integrity [65] and refers to the directionality of diffusion, while radial diffusivity, which refers to diffusion perpendicular to the main diffusion direction, may be more sensitive to demyelination [66]. Mean diffusivity reflects the total level of diffusion in a particular voxel or region. Decreased fractional anisotropy [65], and increased radial diffusivity [66] or mean diffusivity [67] reflect WM pathology. The DTI images were preprocessed with FMRIB Software Library’s (FSL, version 4.1.2 http://www.fmrib.ox.ac.uk/fsl) [68] FDT 2.0 [69]. The DTI images were motion and eddy current corrected. BET [70] was used to produce a mask to include only voxels inside the brain in the diffusion tensor fitting. The diffusion tensors were calculated with DTIFIT. This resulted in images of fractional anisotropy, mean diffusivity, and radial diffusivity (calculated as the mean of the second and third eigenvalues). These images were then processed using Tract-based spatial statistics [71]. The fractional anisotropy images were nonlinearly registered to the FMRIB58 fractional anisotropy standard space, followed by an affine transform to the MNI152 space. They were merged into an average WM tract template (i.e. a ‘skeleton’) with voxel size of 1x1x1 mm and a threshold value of 0.2. The individual fractional anisotropy images were projected onto this skeleton. The same registration vectors were then applied to the mean diffusivity and radial diffusivity images.

### Statistical analyses

We compared the levels of inflammatory and metabolic markers between cases and controls with Mann-Whitney *U*-test; a nonparametric test was used because most of the markers had skewed distributions. Comparisons in sociodemographic and clinical variables were done with the Mann-Whitney *U*-test for continuous variables, and with Pearson’s χ^2^ test, or Fisher’s exact test for categorical variables. For calculating correlations between inflammatory, metabolic and clinical variables, Spearman’s rank order correlation was used for correlations between two continuous variables and Kendall’s Tau for correlations between continuous and binary variables. Paired samples *t*-test was used to compare serum marker levels at baseline and at 2 months. For descriptive purposes, we present all *p*-values significant at the <.05 level. For differences in inflammatory and metabolic markers between cases and controls, also Bonferroni-corrected results are presented. Effect sizes for the between-group differences in serum marker levels were calculated using Hedges’ g with 95% confidence intervals.

To further evaluate the specificity of the associations between serum marker levels and FEP, we made general linear models with the following independent variables, selected on the basis of an analysis of correlations and previous literature: case vs. control status, sex, body mass index (BMI) and current smoking. The analyses were performed using the SAS 9.3 for Windows Software.

The associations of FEP-related inflammatory markers with GM and WM volume were analysed within the patient group with multiple regression analysis within the general linear model framework, where the effects of age, sex and scanner were controlled for. Statistical tests for the volumetric measures were corrected for multiple comparisons according to family-wise error rate (FWE) in SPM8 at single-voxel or cluster level. Associations between GM decrease and the blood measures were corrected for multiple comparisons in the whole brain volume due to numerous loci of GM changes in recent meta-analyses [29,30]. For the WM volume, we corrected findings for multiple comparisons in the whole brain volume and in the volume of four regions of interest (ROIs) that were defined on the basis of a meta-analysis [31]. The ROIs were modelled as spheres with 20 mm radius that were centered at the coordinates of the most frequently reported fractional anisotropy changes and at the corresponding coordinates of the contralateral hemisphere: *x* = -12/12, *y* = 34, *z* = 10 and *x* = -30/30, *y* = -32, *z* = -2. To restrict the amount of DTI analyses, we focused, in addition to the whole-brain search, only on the ROIs where significant associations with WM morphology were found in the volumetric analyses. For the statistical analyses of DTI images, FSL was used and the diffusion values as well as the variables entered into the design matrix, that is, the serum marker level with age, sex and scanner controlled for, were demeaned and threshold-free cluster enhancement-based [72] method with 5000 permutations was used to correct for multiple comparisons. The results of DTI analyses were localized according to the Johns Hopkins University ICBM-DTI-81 WM labels atlas [73] included in FSL.

### Quality analysis of combining data from two scanners

A quality analysis was conducted to assess the reliability of combining data from two separate scanners. For this purpose, we analysed all results separately for the scanners and calculated ROI-specific voxel-wise coefficients of variation (CV), i.e. the ratio of the standard deviation to the mean, as well as voxel-wise intra-class correlation coefficients (ICC) [74] between the scanners, using data from seven healthy controls who had been scanned with both scanners. The ICCs concerning the WM volume between scanners were high (left frontal: 0.90 ± 0.07; right frontal: 0.84 ± 0.11; left temporal: 0.66 ± 0.2; right temporal: 0.76 ± 0.15), which justifies the combination of the data sets from different scanners. The mean CV for WM volume within left frontal ROI was 9.5%, right frontal ROI was 8.4%, left temporal was 14.3% and right temporal ROI was 10.9%.

The CV is a commonly used measure of DTI reproducibility [75]. Therefore, mean CVs of skeletonized fractional anisotropy, mean diffusivity and radial diffusivity maps within both frontal ROIs were computed. The mean CV for fractional anisotropy within left and right frontal ROIs, for radial diffusivity within left and right frontal ROIs and for mean diffusivity within left and right frontal ROIs were: 8.0%, 7.6%, 7.6%, 7.1%, 15.2%, and 14.0%, respectively. It was thus concluded that the DTI signals were variable within the ROIs between scanners and therefore we report the DTI analysis separately for the Siemens scanner (with a larger subsample; *n* = 18) in addition to pooled data from both scanners.

## Results

### Characteristics of the participants

By February 2013, 37 cases and 19 controls had given a blood sample and were included (see description in Table 1). Of them, T1 images for 36 and DTI images for 34 cases were available. The cases and controls were similar in terms of potential confounding factors of age, sex, BMI, self-reported physical activity, and AUDIT score but differed significantly in current smoking, substance abuse as well as in several variables describing functioning and symptoms (Table 1). Hospitalization during the acute phase was needed for 30/37 (81.1%) of the patients. The majority of patients had had antipsychotic medication at baseline assessment, with a median duration of 27 days (from 8 to 68 days). Three patients had had small dose antipsychotic medication for 122, 293, and 717 days, with indications other than psychotic symptoms. The treatment was naturalistic and included switches and combinations of medications. The used antipsychotics were: risperidone (*n* = 10), quetiapine (*n* = 8), olanzapine (*n* = 13), aripiprazole (*n* = 2), chlorpromazine (*n* = 1), sertindole (*n* = 1), haloperidol (*n* = 1), ziprasidone (*n* = 1), perphenazine (*n* = 2) and chlorprotixene (*n* = 1). Sum score of positive psychotic symptoms during the last week at baseline was median 7 (range 0 to 15), and of negative symptoms median 7 (range 0 to 13). Worst episode BPRS score of positive symptoms was median 11 (range 5 to 17).

### Differences in inflammatory and metabolic markers

Serum level of CCL22 was statistically significantly higher in cases than controls and the levels of TGFα, CXCL1, CCL7, IFN-α2 and ApoA-I were lower in cases (Table 2). The significantly increased CCL22 levels in cases were also seen after Bonferroni correction for multiple testing for inflammatory markers (38 tests, corrected α level 0.0013). Other differences in immunological markers or metabolic markers (10 tests, corrected α level 0.005) did not remain significant after Bonferroni correction. The effect sizes (Hegdes’ g) for group differences in markers with approximately normal distribution were 0.92 (95% CI 0.35–1.50) for CCL22, -1.05 (95% CI -1.63– -0.46) for CXCL1 and -0.78 (95% CI -1.35– -0.21) for ApoA-I. While CCL22 levels did not correlate significantly with the other markers, CXCL1 correlated significantly with TGFα (Spearman’s rho = .43, *p* <. 001) and ApoA-I (rho = .27, *p* = .047), CCL7 with IFN-α2 (rho = 0.73, *p* <. 001) and TGFα (rho = 0.49, *p* <. 001) and IFN-α2 with TGFα (rho = 0.34, *p* = .01). In addition, CCL22 was higher in men, CXCL1 was lower in current smokers, and TGFα correlated positively with BMI (S3 Table).

In patients, TGFα correlated with BMI (rho = .35, *p* = .037), and CCL22 with waist circumference (rho = .37, *p* = .04) and BMI (rho = .34, *p* = .045). No statistically significant correlation with inflammatory markers was seen for any symptom scores (S4 Table). Duration of antipsychotic treatment did not correlate with any of the inflammatory or metabolic measures.

In general linear models including case vs. control status, age, sex, smoking and BMI as explanatory variables, case vs. control status remained a significant predictor for CCL22, CXCL1 and ApoA-I. We could not do similar analysis for IFN-α2, TGFα, and CCL7 because of their skewed distribution and the presence of several marked outliers. Instead, we did logistic regression analyses using the same dependent variables and grouping the values of the cyto/chemokines based on the median of the control group as above the median of the control group vs. lower. In these analyses, case-control status did not remain a significant predictor. See S1 Results for a detailed description of the results.

### Changes in inflammatory markers from baseline to 2 months follow-up

Among the inflammatory markers associated with FEP, the level of CCL22 was the only marker that showed a significant change from baseline to 2 months follow-up. The level of CCL22 decreased during follow-up (paired samples *t*-test *t* = -2.2, *df* = 25 *p* = .04), although it was still higher in cases at 2 months than in controls at baseline (mean cases at 2 months vs. controls at baseline 1787.5, SD = 514.3 vs. 1346.9, SD = 398.4, *t* = 3.1, *df* = 43, *p* = .03). Patients had higher BMI (mean 24.4, SD = 4.8); paired samples *t*-test *t* = 3.1, *df* = 25 *p* = .005) and waist circumference (90.0 cm, SD = 2.3; paired samples *t*-test *t* = -4.3, *df* = 28 *p* <. 001) at 2 months follow-up than at the baseline. No other statistically significant changes were detected in metabolic measures.

### The association of psychosis-related inflammatory and metabolic markers with brain measures in patients

**Fig 1:**
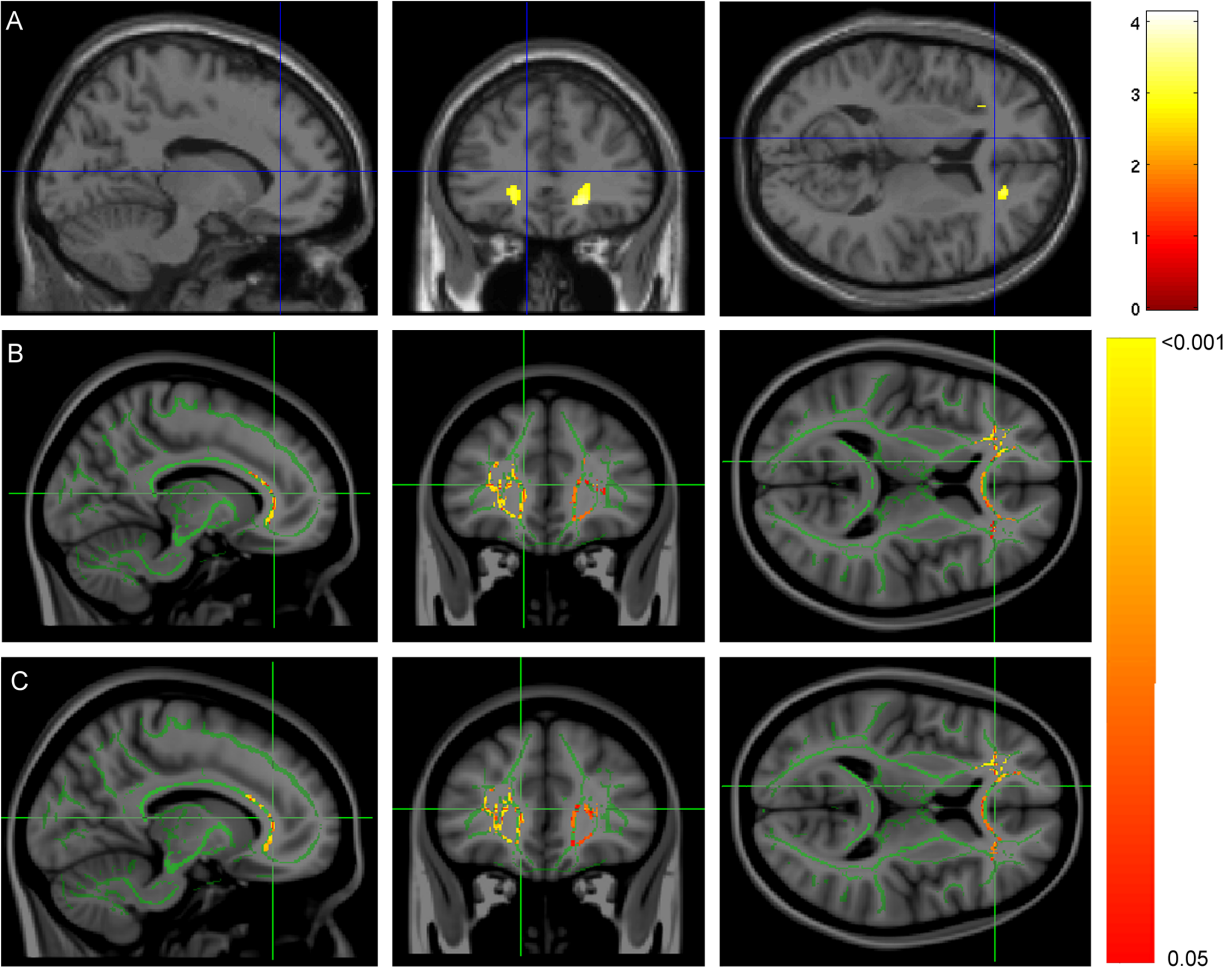
The associations of serum CCL22 levels with white matter volume (WMV) and diffusion measures within the patient group. **(A)** CCL22 level correlated negatively with WMV within the frontal regions of interest (ROIs) (see main text for ROI definitions) bilaterally. Voxels with *p* < 0.005 (uncorrected, for visualization only; see corrected *p*-values in Table 4) within frontal ROIs are shown in hot colors on an SPM’s canonical single subject T1 image. Color bar for the *t*-values depicted in **(A)** is shown on the right. In **(B–C)**, the FMRIB58 FA mean skeleton is shown in green on a T1 template image. **(B)** Mean diffusivity and **(C)** radial diffusivity were positively correlated with CCL22 levels; clusters with *p* < 0.05 TFCE-corrected for family-wise error rate within unilateral ROIs are shown. On the right of **(B)** and **(C),** a color bar shows the corrected *p*-level for these images. Crosshair in all the images is at *x* = -12, *y* = 34, *z* = 10. In the images, left hemisphere is on the left.

**Fig 2:**
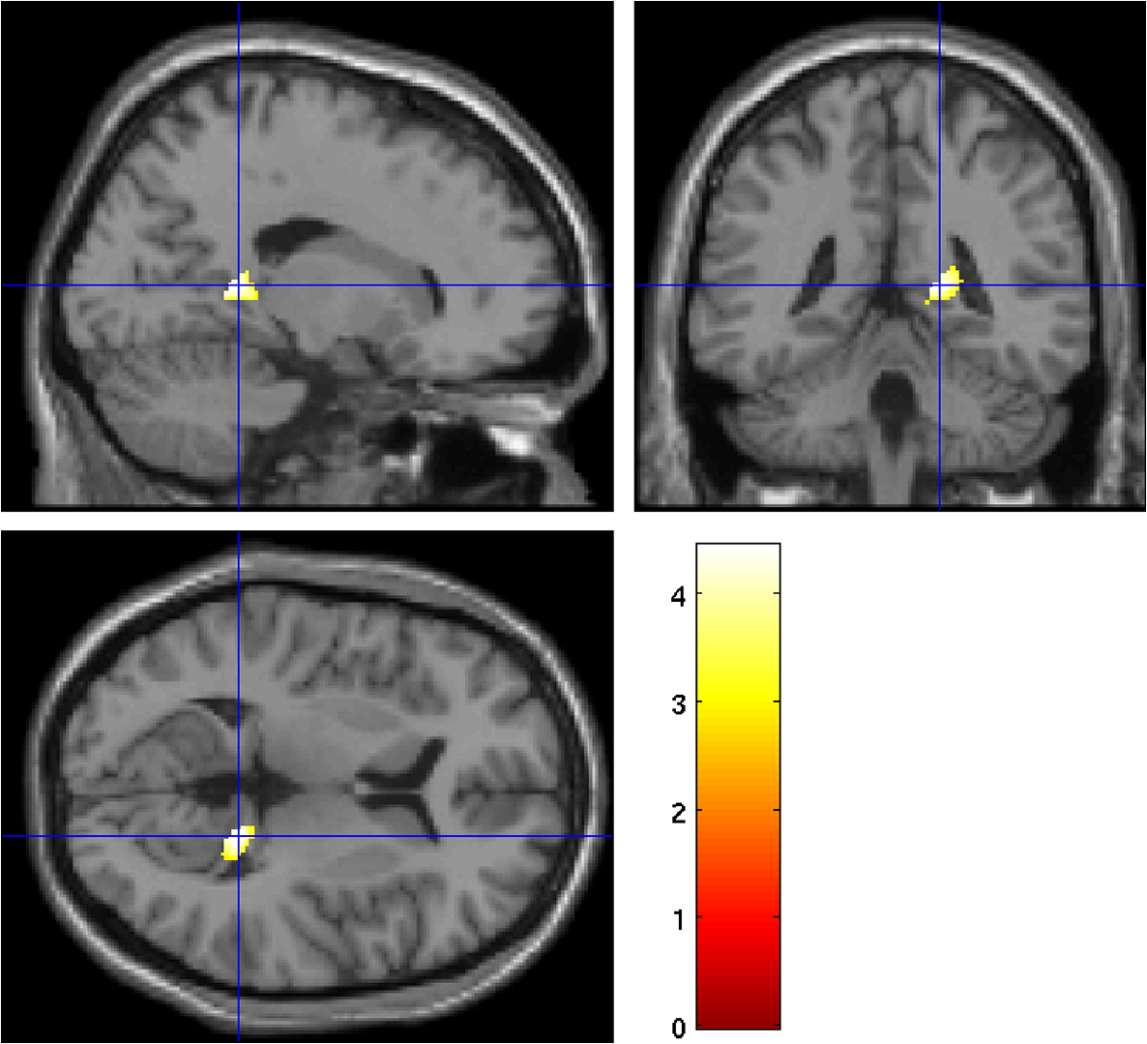
A positive correlation with serum levels of apolipoprotein A-I and white matter volume in right temporal ROI was observed within patients. Voxels with *p* < 0.005 (uncorrected, for visualization only; see corrected *p*-values in Table 4) within the ROI are shown in hot colors on an SPM’s canonical single subject T1-image. Depicted are the sagittal, coronal, and axial views at *x* = 18, *y* = -42, *z* = 7. At the bottom right, a color plate shows the *t*-value. Left hemisphere is on the left.

We then investigated whether the serum levels of markers CCL22, CXCL1, and ApoA-I correlated with brain volume and diffusion measures in FEP patients. The results of these analyses are presented in Table 4. In the ROI analyses of volumetric measures, CCL22 was negatively correlated with WM volume in the left and right frontal lobes (Fig 1A). Decreased serum levels of ApoA-I in patients associated with smaller volume of the medial temporal WM (Fig 2). No statistically significant associations were found between any of these measures and GM volume.

**Table 4:** White matter volume and DTI measures within the patient group associating with inflammatory measures.

Marker	Measure^a^	Peak MNI Coordinates			Extent^b^ (mm^3^)	*p* ^c^	Tracts overlapping with significant clusters (the size of overlap in mm^3^)^d^
		***x***	***y***	***z***			
CCL22	WMV (left)	-20	27	-5	1168	0.044	
	WMV (right)	16	40	15	2298	0.008	
	MD (whole)	-26	31	11	31221	0.009	Multiple tracts^e^
	RD (whole)	-24	32	16	15522	0.015	Multiple tracts^e^
		-48	-39	12	4578	0.035	
		-31	-62	36	19	0.049	
	MD (left)	-26	31	11	2069	0.003	Genu of CC (656.00), body of CC (12.00), left anterior CR (760.00)
	RD (left)	-24	26	11	1874	0.003	Genu of CC (557.00), body of CC (5.00), left anterior CR (756.00)
	MD (right)	-5	30	6	1145	0.015	Genu of CC (604.44), anterior limb of right IC (2.13), right anterior CR (409.17)
		27	29	17	157	0.042	
	RD (right)	-5	30	6	638	0.022	Genu of CC (581.78), right anterior CR (379.59)
		27	33	10	404	0.021	
CXCL1	FA (whole)	-20	-21	42	5053	0.027	Multiple tracts^e^
	MD (whole)	12	20	21	12287	0.029	Multiple tracts^e^
		-16	-15	34	9488	0.018	
	RD (whole)	-20	-17	37	22729	0.008	Multiple tracts^e^
		3	-8	11	413	0.041	
		43	-24	-2	407	0.041	
		21	13	-16	211	0.048	
		23	18	12	132	0.048	
ApoA-I	WMV (right)	18	-42	7	884	0.037	

^a^In the Measure column, left and right refer to the side of the region of interest. Analyses indicated as “whole” are corrected for family-wise error rate for the whole WM tract skeleton volume.

^b^In the case of white matter volume (WMV), extent refers to contiguous voxels with *p* < 0.005, uncorrected, while with the DTI measures, extent refers to clusters defined as in [72].

^c^The results are corrected for family-wise error rate for the whole brain for CCL22 and CXCL1, and for a sphere with a radius of 20 mm for CCL22 (cluster level with a primary threshold of *p* < 0.005) and ApoA-I (peak level). In the DTI analyses, the reported *p*-level is the minimum *p*-level within a cluster.

^d^The tracts and the sizes of overlap are based on the Johns Hopkins University ICBM-DTI-81 WM labels atlas [73] and are a combination of the clusters concerning particular marker and measure. Notice that the sizes of the overlap do not equal the summed extent due to unspecified areas in the atlas.

^e^See S3 Results for full listings of these tracts.

Abbreviations: ApoA-I, apolipoprotein A-I; CC, corpus callosum; CCL, chemokine (C-C motif) ligand; CR, corona radiata; CXCL, Chemokine (C-X-C motif) ligand; FA, fractional anisotropy; IC, internal capsule; MD, mean diffusivity; MNI, Montreal Neurological Institute; RD, radial diffusivity; WMV, white matter volume.

In the whole-brain DTI analysis, CCL22 level was associated with mean diffusivity and radial diffusivity in frontal, parietal, temporal, and occipital lobes. In the ROI analysis of DTI data, CCL22 level correlated positively with mean diffusivity and radial diffusivity bilaterally in the frontal lobes (Fig 1B and 1C). The association of CCL22 and mean diffusivity in the whole-brain analysis, as well as the associations in the ROI analyses, appeared to be robust because they were not affected by the scanner used; the associations were found both in the whole patient group scanned with two different scanners and also in the subgroup of patients (*n* = 18) scanned with the newer Siemens scanner (S5 Table; see also S2 Results).

In the whole-brain analysis, CXCL1 levels associated negatively with fractional anisotropy and positively with mean diffusivity and radial diffusivity, contrary to our hypothesis. These results were reproduced within the subgroup scanned with the Siemens scanner (S5 Table).

## Discussion

The most interesting findings in our study related to the role of chemokine CCL22 in FEP. Circulating CCL22 level was higher in FEP patients than in controls. Furthermore, in FEP patients, a higher level of CCL22 associated with reduced frontal WM volume, as well as diffusion measures previously linked to demyelination [66] and WM pathology [67]. The associations with diffusion measures were mainly located in the genu of corpus callosum and bilateral anterior corona radiata; however, in a whole-brain DTI analysis, CCL22 had more widespread associations in addition to frontal areas.

CCL22, or macrophage-derived chemokine (MDC), acts as a chemo-attractant for chemokine receptor 4 expressing monocytes, dendritic cells, NK cells, B-cells, and T-cell subsets, particularly Th2 cells and regulatory T-cells [76–78]. Previous studies have found that serum level of CCL22 is elevated in chronic schizophrenia [79], predicts relapse in schizophrenia [80], and differentiates schizophrenia from major depressive disorder [81]. In chronic bipolar patients, decreased CCL22 mRNA expression in lymphocytes has been found in comparison to patients with chronic schizophrenia [82]. CCL22 has also been associated with non-psychotic neuropsychiatric diseases, including temporal epilepsy [83], autism [84], several forms of encephalitis [85,86], and multiple sclerosis [87]. In experimental autoimmune encephalomyelitis (EAE), an animal model of multiple sclerosis caused by immunization of animals with whole myelin or myelin products, CCL22 has a key role in disease progression. CCL22 contributes to immune-cell recruitment across the blood-brain barrier, and in the disease remission the increased levels are normalized [86,88,89].

In contrast to CCL22, the levels of CXCL1 and CCL7, produced largely by the same cell types as CCL22, were decreased in FEP. Some earlier reports support an association of CXCL1 and CCL7 with psychotic disorders, although in previous reports their serum level or gene expression has been elevated in patients with schizophrenia [79,80,90,91].

The enhanced production of CCL22 in the patients can be considered as a marker of the activation of Th2 immunity. Th2 type cytokines, such as IL-4 and IL-13, up-regulate the production of CCL22, whereas Th1 type cytokines, such as IFN-γ, down-regulate it [92]. Production of CCL22 is dependent on the phosphorylation of transcription factor STAT6 as shown in STAT6 knock-out mouse model [93]. In contrast, the expression of several other chemokines, such as CXCL1 and CCL7, was increased in STAT6 deficient mice, indicating that STAT6 acts as a negative regulator of these chemokines [93]. Accordingly, decreased levels of CXCL1 and CCL7 in FEP could be explained by the reciprocal effect of STAT6 on these two chemokines versus CCL22. Altogether, the observed profile of systemic chemokines and cytokines in FEP, such as increased CCL22 and decreased CXCL1, CCL7 and IFN-α2, could reflect up-regulation of IL-4 mediated STAT6 signaling [94]. Interestingly, STAT6 is among the immunological genes that were significantly associated with schizophrenia in the largest genome-wide association study of schizophrenia published to date [12]. Also some earlier studies in schizophrenia [95,96] and in FEP [97] suggest activation of Th2 immunity. In FEP, Th2 immunity has been reported to be attenuated after antipsychotic treatment [20]. Interestingly, we found that the CCL22 levels decreased in the patients during the 2-month follow-up, although remained increased compared to the controls.

We applied both volumetric and diffusion measures as indications of WM pathology. It has been suggested that diffusion parameters are more sensitive to WM changes than volumetric measures [98] but both provide information regarding the structural integrity of WM [99]. Although conventional DTI parameters, such as fractional anisotropy and mean diffusivity, are the most applied markers of WM structure and pathology in human neuroimaging [65], the exact biological mechanism behind the neuropathology is not well known. These markers are sensitive to myelination, axonal degeneration, local fiber count and orientation [100], as well as changes in extracellular space which could be more indicative of neuroinflammation [44]. Studies that have modelled the extracellular volume and within-tissue fractional anisotropy separately have suggested increase in extracellular volume (a sign of neuroinflammation) in FEP [44]. However, our parameters do not enable the separation of these etiologies.

Peripheral inflammation has been associated with WM microstructural changes in some earlier studies in healthy, middle-aged individuals [36–38] and with GM volume in mood disorders [35]. Peripheral inflammation could contribute to changes in WM and/or GM by triggering an inflammatory response in the microglia [101]. There is evidence of microglial activation in schizophrenia, and it has been hypothesized that a prenatal infection may leave subsets of microglia permanently in an activated state, and a subsequent immune challenge in adulthood causes exaggerated response in the primed microglial cells [102,103]. CCL22 is potentially an interesting biomarker for WM damage, since in the mouse EAE model it is linked to progressive WM pathology [86,88,89]. Although EAE is used as an animal model for multiple sclerosis, mice in the early stages of EAE show anxiety- and depression-like behavior, social avoidance and memory impairment [104], features commonly seen in genetic mouse models of schizophrenia [105]. However, while our findings are intriguing, more research is needed to unravel the longitudinal process leading to WM damage in schizophrenia, and the role of different immunological mechanisms in it.

The only significant difference in metabolic markers was decreased concentration of ApoA-I, the major structural protein of HDL-C, in FEP patients. A previous study found a consistent decrease in ApoA-I levels in the serum, CSF and post-mortem brain and liver tissue in schizophrenia; the decrease was not explained by confounding factors, such as the use of antipsychotics, and was thus suggested to be linked to the underlying disease mechanisms [106]. We observed an association of decreased serum ApoA-I and smaller volume of the medial temporal WM in FEP patients. Previous studies in healthy adults have found no influence of ApoA-I on WM microstructure [36,37], but an association between HDL-C and GM volume [107]. However, in late-life depression, low ApoA-I is associated with mild cognitive impairment and structural brain changes [52]. The role of ApoA-I may also be associated with inflammation because ApoA-I was shown to attenuate neuroinflammation in a mouse model of Alzheimer’s disease [108].

Although CXCL1 was decreased in patients, higher CXCL1 in patients correlated with changes in WM diffusion. Previously, CXCL1 has been associated with radiologically confirmed infarction after acute ischemic stroke [109] and MS [110].

The limitations of our study include a relatively small size of the sample, with heterogeneity in medication and diagnosis, and multiple testing. In particular, it is possible that due to lack of power we found no significant association between immune markers and GM, or differences in some cytokines suggested as state or trait markers of psychosis [1]. However, the timing of the sampling and the differences in the age of the patients may more likely explain these discrepant findings. Patients with FEP in our study were relatively young and did not suffer from obesity or metabolic comorbidities, which contribute to the peripheral inflammatory markers reported in chronic patients with psychotic disorders [5,14].

A strength of our study was the population-based control group, namely controls matched by age, sex and region of residence selected from the population registry. The selection of the controls may affect the findings significantly. For example, in the study by Dimitrov et al. [79] the same MILLIPLEX Kit as in our study was used and the serum levels of several cytokines and chemokines differ markedly between the control samples in our study and theirs, while the differences in the patient groups are smaller. To avoid the storage-related changes or the effects of circadian and postprandial variation in the chemokine and cytokine levels [111] we collected fasting samples taken in the morning and stored immediately at -80°C. We also analyzed the potential role of confounding factors in the differences found between FEP patients and controls, most importantly physical activity, metabolic factors, duration of medication, and illegal drug use. Furthermore, we present a careful analysis of the potential effect of the use of two scanners, but, after careful quality check, the use of two scanners for brain imaging did not affect the main results of serum markers associating with brain measures.

## Conclusions

In our cohort, FEP patients showed an altered profile of systemic inflammatory markers produced by the innate immune system. The most striking finding was the elevated serum CCL22 level, which showed an association with WM volume and diffusion in WM tracts in FEP. To our knowledge, this is the first study to report an association between peripheral inflammation and WM volume and diffusion in WM tracts in FEP, while activation of the mononuclear phagocyte system in psychosis [2] and alterations in WM regions in FEP and schizophrenia have been demonstrated [30–32]. Our findings support the view that immunological factors can contribute to white matter abnormalities in FEP [40].

## Supporting Information

S1 Results General linear models and logistic regression. (DOCX)

S2 Results Cross-checking DTI results from scanner subsamples. (DOCX)

S3 Results Full listings of white matter tracts overlapping with clusters of significant association. (DOCX)

S1 Table Evaluation of the cohort at baseline and at two months. (DOCX)

S2 Table The minimum detectable concentrations of cytokines/chemokines. (DOCX)

S3 Table Correlations of confounding factors and symptom scores with the main finding cytokines in total sample (cases and controls). (DOCX)

S4 Table Spearman rank order correlations between symptom scores and psychosis-related serum markers in patients at baseline. (DOCX)

S5 Table DTI measures within a subsample (*n* = 18) of patients scanned with the Siemens scanner correlating with serum markers. (DOCX)
