# Supplementary material for: Altered Activation of Innate Immunity Associates with White Matter Volume and Diffusion in First-Episode Psychosis
Source: PLoS One. 2015 May 13;10(5):e0125112. doi: 10.1371/journal.pone.0125112 (PMC4430522; doi:10.1371/journal.pone.0125112)
Supplement: S1 Results — (DOCX) [file pone.0125112.s001.docx]

**S1 Results. General linear models and logistic regression**

General linear models were performed with PROC GLM in SAS. Case-control status, sex, current smoking and BMI were used as independent variables in all the models. Normal distribution of the residuals was checked with the Shapiro-Wilk test.

In the model for CCL22, case-control status (*p* = .005) was a significant predictor, whereas sex (*p* = .24), BMI (*p* = .16) and smoking (*p* = .61) were not. R^2^ for the model was 0.29, and Shapiro-Wilk’s test indicated that the residuals were normally distributed (W = 0.98, *p* = .73).

In the model for CXCL1, case-control status (*p* = .0026) was a significant predictor, whereas BMI (*p* = .07), sex (*p* = .30) and smoking (*p* = .44) were not. R^2^ for the model was 0.34, and Shapiro-Wilk’s test indicated that the residuals were normally distributed (W = 0.97, *p* = .18).

In the model for ApoA-I, case-control status (*p* = .02) and sex (*p* = .02) were significant predictors, whereas BMI (*p* = .86) and smoking (*p* = .88) were not. R^2^ for the model was 0.24. Shapiro-Wilk’s test indicated slight deviation from normal distribution in the residuals (W = 0.93, *p* = .007), but graphical inspection of the residuals did not reveal marked outliers.

Similar analysis was not done for IFN-α2, TGF-α, and CCL7 because of the skewed distribution of these cyto/chemokines and the presence of some marked outliers.

We could not do similar analysis for IFN-α2, TGF-α, and CCL7 because of the skewed distribution of these cyto/chemokines and the presence of several marked outliers. Instead, we did logistic regression analyses. We grouped the values of the cyto/chemokines based on the median of the control group into two groups: above the median vs. lower. The same independent variables were used in these analyses as in the general linear models. Case-control status did not remain a significant predictor in any of the models (*p* = .08 for TGF-α, *p* = .30 for IFN-α2 and *p* = .10 for CCL7).
