# Supplementary material for: Altered Activation of Innate Immunity Associates with White Matter Volume and Diffusion in First-Episode Psychosis
Source: PLoS One. 2015 May 13;10(5):e0125112. doi: 10.1371/journal.pone.0125112 (PMC4430522; doi:10.1371/journal.pone.0125112)
Supplement: S2 Results — (DOCX) [file pone.0125112.s002.docx]

**Supplementary Results S2: Cross-checking DTI results from scanner subsamples**

The positive association between CCL22 levels and frontal MD and RD, as well as whole-brain corrected MD, found in the total patient sample (*n* = 34), was reproduced within the subsample scanned with the Siemens scanner (*n* = 18), although with slightly different cluster extents (Supplementary Table S5). As a further cross-validation method between the scanners, we extracted the mean of MD and RD values from the GE dataset (*n* = 16) corresponding to the cluster showing the most significant correlation in the ROI analyses with the Siemens dataset. A positive one-tailed correlation with CCL22 levels was found between MD and RD values in the right frontal lobe (Spearman’s rho = .483, *p* = .029, and .429, *p* = .048, respectively) and MD values in the left frontal lobe (.475, *p* = .031). The RD values on the left frontal lobe did not reach a significant correlation (.338, *p* = .10) with the GE sample alone.
