# Supplementary material for: Altered Activation of Innate Immunity Associates with White Matter Volume and Diffusion in First-Episode Psychosis
Source: PLoS One. 2015 May 13;10(5):e0125112. doi: 10.1371/journal.pone.0125112 (PMC4430522; doi:10.1371/journal.pone.0125112)
Supplement: S3 Results — (DOCX) [file pone.0125112.s003.docx]

**Supplementary Results S3: Full listings of white matter tracts overlapping with clusters of significant association**

The full lists of white matter tracts where significant associations, as shown in Table 4 of main text, between serum level markers and diffusion measures found in a whole-brain analysis in the pooled data are as following (the size of overlap in mm^3^ in brackets represent the overlap with tracts according to Mori *et al.* [2005]; abbreviations are the same as in Supplementary Table S5):

*CCL22 correlated positively with MD in the following tracts:* Genu of CC (1253.00), body of CC (761.00), splenium of CC (1298.00), left CP (5.00), anterior limb of right IC (92.00), anterior limb of left IC (83.00), posterior limb of right IC (122.00), posterior limb of left IC (231.00), retrolenticular part of right IC (126.00), retrolenticular part of left IC (83.00), right anterior CR (973.00), left anterior CR (1110.00), right superior CR (484.00), left superior CR (666.00), right posterior CR (311.00), left posterior CR (382.00), right posterior thalamic radiation (include optic radiation) (640.00), left posterior thalamic radiation (include optic radiation) (598.00), right sagittal stratum (include ILF and IFOF) (326.00), left sagittal stratum (include ILF and IFOF) (184.00), right EC (460.00), left EC (232.00), right cingulum (cingulate gyrus) (231.00), left cingulum (cingulate gyrus) (56.00), right fornix (cres) / stria terminalis (can not be resolved with current resolution) (5.00), right SLF (572.00), left SLF (704.00), right SFOF (could be a part of anterior IC) (58.00), left SFOF (could be a part of anterior IC) (40.00), right UF (2.00), left UF (24.00), right tapetum (10.00).

*CCL22 correlated positively with RD in the following tracts:* Genu of CC (1203.00), body of CC (801.30), splenium of CC (816.44), left CP (3.25), anterior limb of right IC (9.75.00), anterior limb of left IC (253.38), posterior limb of left IC (185.16), retrolenticular part of right IC (5.41), retrolenticular part of left IC (78.68), right anterior CR (882.49), left anterior CR (1182.43), right superior CR (320.50), left superior CR (137.52), right posterior CR (223.06), left posterior CR (140.77), right posterior thalamic radiation (include optic radiation) (619.37), left posterior thalamic radiation (include optic radiation) (423.74), right sagittal stratum (include ILF and IFOF) (201.40), left sagittal stratum (include ILF and IFOF) (122.72), right EC (387.65), left EC (332.42), right SLF (360.58), left SLF (27.43), left SFOF (could be a part of anterior IC) (45.48), right UF (15.16), right tapetum (15.16), left tapetum (0.72).

*CXCL1 correlated negatively with FA in the following tracts:* Body of CC (1.00), splenium of CC (3.00), left CP (54.00), anterior limb of left IC (32.00), posterior limb of left IC (322.00), retrolenticular part left of IC (219.00), left anterior CR (364.99), left superior CR (543.00), left posterior CR (260.00), left posterior thalamic radiation (include optic radiation) (192.00), left EC (274.00), left SLF (390.00), left SFOF (could be a part of anterior IC) (24.00), left tapetum (1.00).

*CXCL1 correlated positively with MD in the following tracts:* Genu of CC (270.41), body of CC (1524.03), splenium of CC (342.01), fornix (column and body of fornix) (121.46), right CP (66.48), left CP (8.95), anterior limb of right IC (216.08), anterior limb of left IC (57.53), posterior limb of right IC (409.14), posterior limb of left IC (248.68), retrolenticular part of right IC (469.23), retrolenticular part of left IC (122.74), right anterior CR (691.70), left anterior CR (450.69), right superior CR (919.28), left superior CR (652.06), right posterior CR (425.76), left posterior CR (227.58), right posterior thalamic radiation (include optic radiation) (513.98), left posterior thalamic radiation (include optic radiation) (105.48), right sagittal stratum (include ILF and IFOF) (283.84), right EC (337.54), left EC (29.41), right cingulum (cingulate gyrus) (48.59), right fornix (cres) / stria terminalis (can not be resolved with current resolution) (132.97), right SLF (503.75), left SLF (406.58), right SFOF (could be a part of anterior IC) (76.71), left SFOF (could be a part of anterior IC) (30.05), right UF (1.29), right tapetum (23.01), left tapetum (0.64).

*CXCL1 correlated positively with RD in the following tracts:* Genu of CC (631.63), body of CC (1487.74), splenium of CC (336.73), fornix (column and body of fornix) (57.14), left CP (96.94), anterior limb of right IC (10.61), anterior limb of left IC (117.35), posterior limb of right IC (16.33), posterior limb of left IC (482.65), retrolenticular part of right IC (41.63), retrolenticular part of left IC (280.61), right anterior CR (253.67), left anterior CR (987.75), right superior CR (761.22), left superior CR (998.97), right posterior CR (188.77), left posterior CR (437.75), right posterior thalamic radiation (include optic radiation) (261.22), left posterior thalamic radiation (include optic radiation) (268.36), right sagittal stratum (include ILF and IFOF) (1.84), left sagittal stratum (include ILF and IFOF) (103.06), right EC (124.49), left EC (609.18), right cingulum (cingulate gyrus) (33.67), left fornix (cres) / stria terminalis (can not be resolved with current resolution) (11.22), right SLF (573.46), left SLF (913.26), right SFOF (could be a part of anterior IC) (35.51), left SFOF (could be a part of anterior IC) (58.16), left UF (17.35), right tapetum (20.41), left tapetum (1.02).

**References:**

Mori S, Wakana S, Nagae-Poetscher LM, van Zijl, Peter C. M. (2005) MRI atlas of human white matter. Amsterdam, The Netherlands: Elsevier
