# Supplementary material for: Altered Activation of Innate Immunity Associates with White Matter Volume and Diffusion in First-Episode Psychosis
Source: PLoS One. 2015 May 13;10(5):e0125112. doi: 10.1371/journal.pone.0125112 (PMC4430522; doi:10.1371/journal.pone.0125112)
Supplement: S1 Table — (DOCX) [file pone.0125112.s004.docx]

**Supplementary Table S1. Evaluation of the cohort at baseline and at two months.**

|  | **Domain** | **Measure** | **Baseline** | **Two months** |
| --- | --- | --- | --- | --- |
| **Questionnaire** | Depressive symptoms | Beck Depression Inventory (BDI) [Beck *et al.*, 1961] | **x** | **x** |
|  | Anxiety | Beck Anxiety Inventory (BAI) [Beck *et al.*, 1988] | **x** | **x** |
|  | Obsessive-compulsive symptoms | Obsessive-Compulsive Inventory – Revised (OCI-R) [Foa *et al*., 1998] | **x** | **x** |
|  | Harmful alcohol use | Alcohol Use Disorders Identification Test (AUDIT) [Babor *et al*., 2001] | **x** | **x** |
|  | Manic symptoms, lifetime | Mood Disorder Questionnaire [Hirschfeld *et al*., 2000] |  |  |
|  | Physical activity, diet and smoking | Standard survey questions from Finnish general population surveys; Fagerström Nicotine Dependence Scale [Heatherton *et al*., 1991] | **x** | **x** |
| **Interview** | Sociodemographic factors | Questions regarding living situation, family, education, occupation, employment | **x** | **x** |
|  | Psychotic symptoms | 24-item version of the Brief Psychiatric Rating Scale (BPRS) [Ventura *et al*., 1993], including the current severity and severity during the worst period before the baseline assessment, complemented by 3 domains (alogia, anhedonia-asociality and avolition-apathy) from the Scale for the Assessment of Negative Symptoms (SANS) [Andreasen, 1982] to allow assessment of remission [Andreasen *et al*., 2005] | **x** | **x** |
|  | Positive symptoms | Sum of BPRS items Hallucinations (10), Unusual thought content (11), Bizarre behavior (12) and Conceptual disorganization (15); BPRS items were rescaled from 1-7 to 0-6 for calculating the sum score | **x** | **x** |
|  | Negative symptoms | BPRS item Blunted affect (16) and SANS items Alogia, Anhedonia and Avolition; BPRS item was rescaled from 1-7 to 0-6 for calculating the sum score | **x** | **x** |
|  | Current manic symptoms | Young Mania Rating Scale (YMRS) [Young *et al*., 1978] | **x** | **x** |
|  | Psychiatric diagnosis | Research version of the Structured Clinical Interview for DSM-IV disorders (January 2007 revision) |  | **x** |
|  | Functioning | Global Assessment of Functioning (GAF), Social and Occupational Functioning Assessment Scale (SOFAS) [APA, 2000; Hilsenroth *et al*., 2000] | **x** | **x** |
|  | Weight, height, blood pressure and waist circumference |  | **x** | **x** |
|  | Family history of psychiatric disorders |  | **x** |  |
| **Assessed based on interview and case records** | Medication | Duration of antipsychotic medication, type of medication, dosage | **x** | **x** |
|  | Substance use | Substance use assessed based on all available data (self-report, case records, laboratory screening) | **x** | **x** |
|  | Psychiatric diagnoses | Evaluated by a senior psychiatrist based on SCID interview and case record data from all lifetime treatment contacts |  | **x** |

*Abbreviations:* AUDIT, the alcohol use disorders identification test; BAI, Beck Anxiety Inventory; BDI, Beck Depression Inventory; BPRS, Brief Psychiatric Rating Scale; OCI-R, Obsessive Compulsive Inventory- Revised Scale; SANS, Scale for the Assessment of Negative Symptoms; SCID-IV-R, the Research Version of the Structured Clinical Interview for the Diagnostic and Statistical Manual of Mental Disorders IV–interview; YMRS, Young Mania Rating Scale.

**References**

American Psychiatric Association. (2000) Diagnostic and statistical manual of mental disorders. Washington, DC: American Psychiatric Press.

Andreasen NC. (1982) Negative symptoms in schizophrenia: Definition and reliability. Arch Gen Psychiatry 39: 784.

Andreasen NC, Carpenter WT Jr, Kane JM, Lasser RA, Marder SR, et al. (2005) Remission in schizophrenia: Proposed criteria and rationale for consensus. Am J Psychiatry 162: 441-449.

Babor TF, Higgins-Biddle JC, Saunders JB, Monteiro MG. (2001) AUDIT - the alcohol use disorders identification test: Guidelines for use in primary care. Geneva: World Health Organization.

Beck AT, Epstein N, Brown G, Steer RA. (1988) An inventory for measuring clinical anxiety: Psychometric properties. J Consult Clin Psychol 56: 893-897.

Beck AT, Ward CH, Mendelson M, Mock J, Erbaugh J. (1961) An inventory for measuring depression. Arch Gen Psychiatry 4: 561-571.

Foa EB, Kozak PM, Salkovskis ME, Coles NA. (1998) The validation of a new obsessive-compulsive disorder scale: The obsessive-compulsive inventory. Psychol Assessment 10: 206-214.

Heatherton TF, Kozlowski LT, Frecker RC, Fagerstrom K. (1991) The fagerstrom test for nicotine dependence: A revision of the fagerstrom tolerance questionnaire. Br J Addict 86: 1119-1127.

Hilsenroth MJ, Ackerman SJ, Blagys MD, Baumann BD, Baity MR, et al. (2000) Reliability and validity of DSM-IV axis V. Am J Psychiatry 157: 1858-1863.

Hirschfeld RMA, Williams JBW, Spitzer RL, Calabrese JR, Flynn L, et al. (2000) Development and validation of a screening instrument for bipolar spectrum disorder: The mood disorder questionnaire. Am J Psychiatry 157: 1873-1875.

Ventura J, Lukoff D, Nuechterlein KH, Liberman RP, Green MF, et al. (1993) Brief psychiatric rating scale (BPRS) expanded version: Scales, anchor points, and administration manual. Int J Method Psych 3: 227-227-243.

Young RC, Biggs JT, Ziegler VE, Meyer DA. (1978) A rating scale for mania: Reliability, validity and sensitivity. Br J Psychiatry 133: 429-435.
