# Supplementary material for: Altered Activation of Innate Immunity Associates with White Matter Volume and Diffusion in First-Episode Psychosis
Source: PLoS One. 2015 May 13;10(5):e0125112. doi: 10.1371/journal.pone.0125112 (PMC4430522; doi:10.1371/journal.pone.0125112)
Supplement: S2 Table — (DOCX) [file pone.0125112.s005.docx]

**Supplementary Table S2. The minimum detectable concentrations of cytokines/chemokines.**

|  | **Measure** | **MinDC** | **n (%) of results below MinDC** |
| --- | --- | --- | --- |
| **Innate immune system markers** | |  |  |
| IFN-α2 | pg/ml | 3,2 | 2 (3.6 %) |
| IL-1a | pg/ml | 3,2 | 19 (33.9 %) |
| IL-1ra | pg/ml | 8,3 | 3 (5.4 %) |
| IL-1β | pg/ml | 0,8 | 23 (41.1 %) |
| IL-6 | pg/ml | 1,28 | 20 (35.7 %) |
| TNF**-**α | pg/ml | 0,7 | 0 |
| **Th1 cytokines** |  |  |  |
| IFN-γ | pg/ml | 1,28 | 0 |
| IL-12p40 | pg/ml | 7,4 | 22 (39.3%) |
| IL-12p70 | pg/ml | 1,28 | 7 (12.5 %) |
| **Th2 cytokines** |  |  |  |
| IL-4 | pg/ml | 4,5 | 17 (30.4 %) |
| IL-5 | pg/ml | 1,28 | 33 (58.9 %) |
| IL-13 | pg/ml | 1,3 | 24 (42.9 %) |
| **Th17 cytokines** |  |  |  |
| IL-17 | pg/ml | 0,7 | 3 (5.4 %) |
| **Regulatory T-cell cytokines** | |  |  |
| IL-10 | pg/ml | 1,28 | 16 (28.6 %) |
| **Other cytokines** |  |  |  |
| IL-2 | pg/ml | 1 | 24 (42.9 %) |
| IL-3 | pg/ml | 1,28 | 29 (51.8 %) |
| IL-7 | pg/ml | 1,4 | 2 (3.6 %) |
| IL-8 | pg/ml | 0,4 | 0 |
| IL-9 | pg/ml | 1,2 | 27 (48.2 %) |
| IL-15 | pg/ml | 1,28 | 26 (46.4 %) |
| **Chemokines** |  |  |  |
| CCL2 | pg/ml | 3,2 | 0 |
| CCL3 | pg/ml | 2,9 | 11 (19.6 %) |
| CCL4 | pg/ml | 3,2 | 0 |
| CCL7 | pg/ml | 3,8 | 8 (14.3 %) |
| CCL11 | pg/ml | 3,2 | 0 |
| CCL22 | pg/ml | 16 | 0 |
| CXCL1 | pg/ml | 9,9 | 0 |
| CX3CL1 | pg/ml | 22,7 | 6 (10.7 %) |
| CXCL10 | pg/ml | 3,2 | 0 |
| **Other inflammation related markers** | |  |  |
| TGFα | pg/ml | 0,51 | 0 |
| EGF | pg/ml | 3,2 | 0 |
| FGF-2 | pg/ml | 16 | 1 (1.8 %) |
| FLT-3L | pg/ml | 5,4 | 32 (57.1 %) |
| G-CSF | pg/ml | 3,2 | 0 |
| GM-CSF | pg/ml | 7,5 | 0 |
| TNF-β | pg/ml | 1,5 | 21 (37.5 %) |
| VEGF | pg/ml | 16 | 0 |
| sCD40L | pg/ml | 16 | 0 |

*Abbreviations:* CCL, chemokine (C-C motif) ligand; CXCL, Chemokine (C-X-C motif) ligand; EGF, epidermal growth factor; FGF, fibroblast growth factor; FLT-3L, Fms-related tyrosine kinase 3 ligand; G-CSF, Granulocyte-colony stimulating factor; GM-CSF, granulocyte-macrophage colony-stimulating factor; HDL-C, high density lipoprotein cholesterol; hs-CRP, high sensitivity C-reactive protein; IFN, interferon; IL, interleukin; LDL-C, Low Density Lipoprotein cholesterol; sCD40L, soluble CD40 Ligand; TGF, transforming growth factor; TNF, tumor necrosis factor.
