# Supplementary material for: Altered Activation of Innate Immunity Associates with White Matter Volume and Diffusion in First-Episode Psychosis
Source: PLoS One. 2015 May 13;10(5):e0125112. doi: 10.1371/journal.pone.0125112 (PMC4430522; doi:10.1371/journal.pone.0125112)
Supplement: S3 Table — (DOCX) [file pone.0125112.s006.docx]

**Supplementary Table S3. Correlations of confounding factors and symptom scores with the main finding cytokines in total sample (cases and controls).**

|  | **CXCL1** | |  | **CCL7** | | |  | **CCL22** | | |  | **IFN-α2** | | |  | **TGFα** | |  | |
| --- | --- | --- | --- | --- | --- | --- | --- | --- | --- | --- | --- | --- | --- | --- | --- | --- | --- | --- | --- |
|  | rho/ tau^a^ | *p* | rho/ tau | | *p* | rho/  tau | | | *p* | rho/  tau | | | *p* | rho/  tau | | | *p* | |  |
| Age | .07 | .61 | -.14 | | .32 | -.19 | | | .15 | -.23 | | | .10 | .05 | | | .73 | |  |
| Female | .19 | .10 | .04 | | .75 | **-.26** | | | **.02** | -.05 | | | .67 | .11 | | | .32 | |  |
| BMI BL | .21 | .13 | .26 | | .06 | .21 | | | .12 | .05 | | | .72 | **.31** | | | **.02** | |  |
| Smoking | .29 | .01 | .13 | | .30 | .20 | | | .10 | .04 | | | .73 | -.21 | | | .07 | |  |
| Substance use lifetime^b^ | -.07 | .55 | -.06 | | .62 | .14 | | | .20 | .06 | | | .57 | .01 | | | .93 | |  |
| Active in sports min. 1h weekly | .22 | .05 | .07 | | .51 | -.12 | | | .28 | .10 | | | .38 | .03 | | | .76 | |  |

^a^Spearman’s rank order correlation (rho) was used for correlations between continuous variables, Kendall’s Tau for correlations between continuous and dichotomous variable

^b^Does not include alcohol, nicotine or caffeine.

*Abbreviations*: BL, baseline; BMI, body mass index; CCL, chemokine (C-C motif) ligand; CXCL, Chemokine (C-X-C motif) ligand; IFN, interferon; rho, Spearman rank order correlation coefficient; TGF, transforming growth factor.
