# Supplementary material for: Altered Activation of Innate Immunity Associates with White Matter Volume and Diffusion in First-Episode Psychosis
Source: PLoS One. 2015 May 13;10(5):e0125112. doi: 10.1371/journal.pone.0125112 (PMC4430522; doi:10.1371/journal.pone.0125112)
Supplement: S4 Table — (DOCX) [file pone.0125112.s007.docx]

**Supplementary Table S4. Spearman rank order correlations between symptom scores and psychosis-related serum markers in patients at baseline.**

HDL-C ApoA-I TGFα IFN-α2 CXCL1 CCL7 CCL22

AUDIT corr. .031 .009 -.356 .167 -.241 -.094 .026

*p*^a^ .876 .963 .063 .395 .216 .636 .897

BDI corr. .269 .200 .005 -.131 .008 -.030 .006

*p*  .150 .290 .981 .489 .965 .875 .973

MDQ corr. .067 .129 .166 .068 -.121 -.048 .355

*p*  .725 .498 .382 .721 .525 .799 .054

BAI corr. .242 .220 .100 .149 -.010 .254 -.029

*p*  .197 .242 .599 .433 .959 .176 .879

OCI-R corr. .438* .362 .184 .016 -.116 .169 .169

*p*  .017 .053 .339 .936 .549 .382 .382

Negative symptoms (Current)

corr. -.053 -.159 -.088 -.236 -.030 -.057 .205

*p*  .757 .346 .603 .160 .859 .737 .223

Positive symptoms (Current)

corr. -.066 .022 .118 .294 -.009 . 281 -.031

*p*  .698 .898 .487 .077 .956 .092 .854

Positive symptoms (Worst)

corr. .001 -.023 .132 .156 .167 .058 .116

*p*  .995 .891 .435 .357 .324 .732 .495

^a^*p-*level statistics are two-tailed.

* significant at *p* < .05.

*Abbreviations*: Apo, apolipoprotein; AUDIT, Alcohol Use Disorders Identification Test; BAI, Beck Anxiety Inventory; BDI, Beck Depression Inventory; CCL, chemokine (C-C motif) ligand; CXCL, Chemokine (C-X-C motif) ligand; HDL-C, high density lipoprotein cholesterol; IFN, interferon; MDQ, Mood Disorder Questionnaire; OCI-R, Obsessive-Compulsive Inventory- Revised Scale; TGF, transforming growth factor.
