# Supplementary material for: Altered Activation of Innate Immunity Associates with White Matter Volume and Diffusion in First-Episode Psychosis
Source: PLoS One. 2015 May 13;10(5):e0125112. doi: 10.1371/journal.pone.0125112 (PMC4430522; doi:10.1371/journal.pone.0125112)
Supplement: S5 Table — (DOCX) [file pone.0125112.s008.docx]

**S5 Table. DTI measures within a subsample (*n* = 18) of patients scanned with the Siemens scanner correlating with serum markers.**

| Marker | Measure | Peak MNI Coordinates | | | | | Extent^a^ (mm^3^) | *p*^b^ | Tracts overlapping with significant clusters (the size of overlap in mm^3^)^c^ |
| --- | --- | --- | --- | --- | --- | --- | --- | --- | --- |
|  |  | *x* | | *y* | | *z* |  |  |  |
| CCL22 | MD (left)^d^ | -18 | 39 | | 17 | | 1235 | 0.02 | Genu of CC (172.31), left anterior CR (441.72) |
|  |  | -20 | 35 | | -1 | | 16 | 0.045 |  |
|  |  | -9 | 29 | | 13 | | 6 | 0.045 |  |
|  |  | 2 | 25 | | 0 | | 1 | 0.046 |  |
|  | RD (left) | -13 | 23 | | 21 | | 1060 | 0.022 | Genu of CC (342.82), body of CC (3.00), left anterior CR (375.85) |
|  |  | -16 | 32 | | 15 | | 2 | 0.050 |  |
|  |  |  |  | |  | |  |  |  |
|  | MD (right) | 2 | 25 | | 0 | | 519 | 0.033 | Genu of CC (202.84), right anterior CR (172.79) |
|  |  | 22 | 37 | | -2 | | 82 | 0.041 |  |
|  | RD (right) | 13 | 32 | | 9 | | 48 | 0.040 | Genu of CC (42.84), right anterior CR (19.16) |
|  |  | 14 | 30 | | 13 | | 14 | 0.043 |  |
|  |  |  |  | |  | |  |  |  |
|  | MD (whole) | 34 | -66 | | 17 | | 15972 | 0.029 | Multiple tracts^e^ |
|  |  | -32 | 35 | | 0 | | 9120 | 0.030 |  |
|  |  | 39 | 8 | | 18 | | 7 | 0.049 |  |
|  |  | 44 | 7 | | 16 | | 4 | 0.049 |  |
| CXCL1 | FA (whole) | -18 | 36 | | 11 | | 7665 | 0.018 | Multiple tracts^f^ |
|  |  | -27 | -22 | | 15 | | 1088 | 0.030 |  |
|  |  | -24 | -49 | | 24 | | 479 | 0.033 |  |
|  |  | 42 | -28 | | 4 | | 100 | 0.045 |  |
|  |  | -45 | -24 | | -2 | | 60 | 0.044 |  |
|  |  | 25 | -50 | | 22 | | 47 | 0.047 |  |
|  |  | -33 | -51 | | 12 | | 11 | 0.050 |  |
|  |  | -32 | -46 | | 13 | | 9 | 0.050 |  |
|  |  | 5 | 26 | | -2 | | 3 | 0.050 |  |
|  |  | 31 | -51 | | 16 | | 2 | 0.050 |  |
|  |  | 8 | 28 | | -3 | | 1 | 0.050 |  |
|  | MD (whole) | -26 | -8 | | 18 | | 39759 | 0.001 | Multiple tracts^g^ |
|  |  | 28 | -61 | | -41 | | 507 | 0.046 |  |
|  |  | 5 | -4 | | 7 | | 134 | 0.049 |  |
|  |  | 13 | -22 | | -1 | | 100 | 0.049 |  |
|  |  | 9 | -50 | | -27 | | 63 | 0.048 |  |
|  |  | 6 | -59 | | -26 | | 22 | 0.049 |  |
|  |  | 28 | -62 | | -36 | | 6 | 0.050 |  |
|  |  | 10 | -14 | | 9 | | 4 | 0.050 |  |
|  |  | -14 | -68 | | -36 | | 4 | 0.050 |  |
|  |  | -17 | -61 | | -39 | | 1 | 0.050 |  |
|  | RD (whole) | -27 | -13 | | 28 | | 44008 | 0.002 | Multiple tracts^h^ |
|  |  | -17 | -60 | | -38 | | 1141 | 0.043 |  |

^a^Extent refers to clusters defined as in [Smith & Nichols, 2009].

^b^The results are corrected for family-wise error rate in the whole brain (whole) or for a sphere with a radius of 20 mm (left/right).

^c^The tracts and the sizes of overlap are based on the Johns Hopkins University ICBM-DTI-81 WM labels atlas [Mori *et al*., 2005] and is a combination of the clusters concerning particular marker and measure. Notice that the sizes of the overlap do not equal the summed extent due to unspecified areas in the atlas.

^d^Left and right refer to the side of the region of interest.

^e^Genu of CC (664.02), body of CC (427.53), splenium of CC (1153.68), anterior limb of right IC (100.31), anterior limb of left IC (24.86), posterior limb of right IC (49.71), retrolenticular part of right IC (177.90), retrolenticular part of left IC (129.96), right anterior CR (633.83), left anterior CR (719.94), right superior CR (150.20), left superior CR (170.44), right posterior CR (151.27), left posterior CR (170.44), right posterior thalamic radiation (include optic radiation) (645.55), left posterior thalamic radiation (include optic radiation) (592.29), right sagittal stratum (include ILF and IFOF) (220.51), left sagittal stratum (include ILF and IFOF) (167.25), right EC (464.28), left EC (145.76), left cingulum (cingulate gyrus) (241.82), right cingulum (hippocampus) (2.13), right fornix (cres) / stria terminalis (can not be resolved with current resolution) (14.91), left fornix (cres) / stria terminalis (can not be resolved with current resolution) (5.33), right SLF (855.41), left SLF (569.92), right SFOF (could be a part of anterior IC) (52.38), right UF (7.46), left UF (11.72), right tapetum (4.26), left tapetum (1.07).

^f^Genu of CC (593.01), body of CC (1206.80), splenium of CC (93.80), left CP (16.85), anterior limb of left IC (255.36), posterior limb of left IC (238.89), retrolenticular part of left IC (218.11), right anterior CR (227.56), left anterior CR (1071.91), right superior CR (95.76), left superior CR (551.82), right posterior CR (5.05), left posterior CR (115.23), right posterior thalamic radiation (include optic radiation) (0.19), left posterior thalamic radiation (include optic radiation) (155.11), left EC (536.75), left cingulum (cingulate gyrus) (35.01), left SLF (662.09), left SFOF (could be a part of anterior IC) (1.03), left UF (4.12), right tapetum (1.31), left tapetum (1.68).

^g^Middle cerebellar peduncle (218.13), genu of CC (395.50), body of CC (1516.76), splenium of CC (1092.15), fornix (column and body of fornix) (21.68), right inferior cerebellar peduncle (24.39), right superior cerebellar peduncle (3.01), right CP (148.56), left CP (102.39), anterior limb of right IC (287.09), anterior limb of left IC (334.27), posterior limb of right IC (536.04), posterior limb of left IC (620.36), retrolenticular part of right IC (571.17), retrolenticular part of left IC (503.92), right anterior CR (822.12), left anterior CR (959.65), right superior CR (1065.04), left superior CR (1188.51), right posterior CR (613.33), left posterior CR (551.09), right posterior thalamic radiation (include optic radiation) (708.69), left posterior thalamic radiation (include optic radiation) (524.99), right sagittal stratum (include ILF and IFOF) (420.60), left sagittal stratum (include ILF and IFOF) (293.11), right EC (602.29), left EC (736.80), right cingulum (cingulate gyrus) (120.46), right cingulum (hippocampus) (1.00), right fornix (cres) / Stria terminalis (can not be resolved with current resolution) (66.25), left fornix (cres) / Stria terminalis (can not be resolved with current resolution) (41.16), right SLF (986.75), left SLF (834.17), right SFOF (could be a part of anterior IC) (60.23), left SFOF (could be a part of anterior IC) (22.08), right UF (6.02), left UF (24.09), right tapetum (19.07), left tapetum (2.01).

^h^Middle cerebellar peduncle (78.49), genu of CC (730.23), body of CC (1643.77), splenium of CC (950.00), fornix (column and body of fornix) (82.04), right CP (168.12), left CP (130.65), anterior limb of right IC (132.68), anterior limb of left IC (428.41), posterior limb of right IC (524.63), posterior limb of left IC (600.59), retrolenticular part of right IC (580.33), retrolenticular part of left IC (414.23), right anterior CR (887.21), left anterior CR (1221.43), right superior CR (997.61), left superior CR (1180.92), right posterior CR (615.78), left posterior CR (604.64), right posterior thalamic radiation (include optic radiation) (821.38), left posterior thalamic radiation (include optic radiation) (627.94), right sagittal stratum (include ILF and IFOF) (384.86), left sagittal stratum (include ILF and IFOF) (248.14), right EC (714.02), left EC (834.54), right cingulum (cingulate gyrus) (53.68), left cingulum (cingulate gyrus) (58.74), right cingulum (hippocampus) (24.31), right fornix (cres) / Stria terminalis (can not be resolved with current resolution) (72.92), left fornix (cres) / Stria terminalis (can not be resolved with current resolution) (55.70), right SLF (929.75), left SLF (938.86), right SFOF (could be a part of anterior IC) (54.69), left SFOF (could be a part of anterior IC) (29.37), right UF (8.10), left UF (23.29), right tapetum (25.32), left tapetum (2.03).

*Abbreviations:* CC, corpus callosum; CCL, chemokine (C-C motif) ligand; CR corona radiata; EC, external capsule; IC, internal capsule; IFOF, inferior fronto-occipital fasciculus; ILF, inferior longitudinal fasciculus; MD, mean diffusivity; MNI, Montreal Neurological Institute; RD, radial diffusivity; SFOF, superior fronto-occipital fasciculus; SLF, superior longitudinal fasciculus; UF, uncinate fasciculus.

**References**

Mori S, Wakana S, Nagae-Poetscher LM, van Zijl, Peter C. M. (2005) MRI atlas of human white matter. Amsterdam, The Netherlands: Elsevier

Smith SM, Nichols TE. (2009) Threshold-free cluster enhancement: Addressing problems of smoothing, threshold dependence and localisation in cluster inference. Neuroimage 44: 83-98.
